# EUROLIGHT project: impact of primary headache disorders from a population-based study conducted in Pavia

**DOI:** 10.1186/1129-2377-1-S14-P209

**Published:** 2013-02-21

**Authors:** M Allena, C Tassorelli, B Carugno, R De Icco, C Andree, G Nappi

**Affiliations:** 1 HSC, Headache Science Centre, IRCCS “National Neurological Institute C. Mondino” Foundation and University of Pavia and UCADH, Italy; 2 ASL, Azienda Sanitaria Locale of Pavia area, Pavia, Italy; 3 CRP, Centre Recherche Publique, Luxembourg, Luxembourg

## Introduction

Headache disorders, including migraine and tension-type headache, are very common in the general population but there is very little recognition of their public health impact [1]. In Italy, there are relatively few studies on the prevalence of primary headaches. We conducted a survey in the population of the Pavia province, in Northern Italy, which is a part of a global project performed at the European Union level, the Eurolight Project (http://www.eurolight-online.eu), to estimate the impact of headaches using a validated tool (the Eurolight questionnaire).

## Material and methods

The Eurolight questionnaire, including 103 items, was distributed to a stratified sample (n=3500) of the adult inhabitants of Pavia province, randomly selected in cooperation with Azienda Sanitaria Locale (ASL). Of these questionnaires, 500 were returned completed correctly.

## Results

487 questionnaires were considered for the analysis (51% by women and 49% by men). Nearly 80% of our study population reported to suffer from episodic headaches in their life and 91.7% had episodic headaches in the last year. Medication Overuse Headache (MOH) was diagnosed in 1.9%. Up to 80.0% of responders suffering from headache never received a diagnosis by a doctor and only 2.4% of them were taking preventative medication. Almost 12% of headache sufferers reported a moderate or severe negative interference of pain in many aspects of the life (education, career and earnings, family planning). Headache influenced the mood state and there was a correlation between the monthly headache frequency and anxiety or depression symptoms. In MOH patients the presence of anxiety and depression disorders was indeed very high.

## Conclusions

Despite a high prevalence of primary headaches in Italian adult population, the majority of affected people are primarily self-treating without receiving the advice of health professionals. Education of patients and health carers should be a high priority issue in public health.
